# EQ-5D-5L population norms for Singapore: a household survey-based analysis

**DOI:** 10.1007/s11136-026-04272-2

**Published:** 2026-06-06

**Authors:** Akemu Xiamusiya, Jia Jia Lee, Yiyun Shou, Shuang Hao, Mythily Subramaniam, Ling Jie Cheng, Nan Luo

**Affiliations:** 1 Department of Learning Informatics, Management and Ethics Medical Management Centre, Karolinska Institutet, Stockholm, Sweden; 2 Saw Swee Hock School of Public Health, National University of Singapore, Singapore, Singapore; 3 Lloyd’s Register Foundation Institute for the Public Understanding of Risk National University of Singapore, Institute of Mental Health, Singapore, Singapore; 4 School of Medicine and Psychology, Australian National University, Canberra, Australia; 5 Department of Medical Epidemiology and Biostatistics, Karolinska Institutet, Stockholm, Sweden; 6 Research Division, Institute of Mental Health, Singapore, Singapore; 7 Lee Kong Chian School of Medicine, Nanyang Technological University, Singapore, Singapore; 8 National Perinatal Epidemiology Unit, Nuffield Department of Women’s & Reproductive Health, University of Oxford, Oxford, UK; 9 Alice Lee Centre for Nursing Studies, Yong Loo Lin School of Medicine National University of Singapore, Singapore, Singapore

**Keywords:** EQ-5D, Population norms, Singapore, Health-related quality of life

## Abstract

**Background:**

Population norms for the EQ-5D-5L are essential for health economic evaluations. However, existing norms for Singapore were established before the COVID-19 pandemic, whose prolonged health and societal impacts may have rendered them outdated. Updated post-pandemic benchmarks are therefore needed to inform cost-utility analyses and public health policy accurately. This study aimed to establish updated EQ-5D-5L population norms among Singapore residents aged ≥ 15 years and to examine variation in health-related quality of life (HRQoL) by sociodemographic characteristics.

**Methods:**

We conducted a cross-sectional household survey between May and July 2024 among Singapore citizens and permanent residents, using a three-stage sampling strategy with demographic quotas. We assessed HRQoL using the EQ-5D-5L and EQ VAS. We derived index scores using the Singapore-specific value set. We generated descriptive statistics and used multivariable linear regression to identify independent associations between HRQoL and sociodemographic characteristics.

**Results:**

The analysis included 2,005 respondents. While 54.6% reported no problems (‘11111’), pain/discomfort (30.7%) and anxiety/depression (27.1%) were the most prevalent issues. The mean EQ-5D-5L index score was 0.933 (SD 0.122), and the mean EQ VAS score was 82.3 (SD 13.3). Multivariable regression showed that older age was most strongly associated with lower HRQoL (*p* < 0.001). Higher socioeconomic status, measured by income and housing type, was independently associated with better HRQoL. Gender was not significantly associated with either outcome.

**Conclusion:**

This study provides updated, post-pandemic EQ-5D-5L population norms for Singapore. These norms offer an essential benchmark for interpreting EQ-5D-5L scores in cost-utility analyses and highlight priorities for addressing socioeconomic health inequalities.

**Supplementary Information:**

The online version contains supplementary material available at 10.1007/s11136-026-04272-2.

## Background

Assessment of health-related quality of life (HRQoL) is a cornerstone of modern health policy and economic evaluation and is essential for allocating scarce healthcare resources efficiently [1]. Quantifying HRQoL enables calculation of quality-adjusted life years (QALYs), the standard outcome measure in cost-utility analyses (CUA) and health technology assessments (HTA) [2]. Among the most widely used preference-based HRQoL measures is the EQ-5D instrument [3].

The EQ-5D is available in more than 150 languages [4] and comprises two components: a descriptive system and the EQ visual analogue scale (EQ VAS). The descriptive system assesses health across five dimensions: mobility, self-care, usual activities, pain/discomfort, and anxiety/depression [5, 6]. The EQ-5D-5L expands the three response levels of the EQ-5D-3L to five, improving sensitivity [7] and reducing ceiling effects [8, 9]. To support economic evaluation, health states defined by the descriptive system are converted into a single utility index using country-specific value sets derived from population preference studies [10, 11].

Population norms serve several key functions. They provide reference points for policymakers to assess population health, monitor inequalities, and track trends over time [12, 13]. They also contextualise clinical trial findings by enabling comparison of patient HRQoL with general population benchmarks [13–15]. Normative EQ-5D-5L data are available for many countries [16–19], including Singapore [20]. However, existing norms, including those reported in 2025, rely on data collected before 2020 and therefore predate the COVID-19 pandemic.

The pandemic constituted an unprecedented global health shock with profound consequences for physical and mental well-being. International evidence shows substantial deterioration in self-reported health, including marked reductions in HRQoL, particularly in the anxiety/depression dimension. Studies report elevated psychological distress among younger adults in the United States [21], persistent post-infection HRQoL deficits in Canada [22], and similar patterns across multinational analyses [23]. In Singapore, recent evidence indicates elevated psychological distress among youth, with 14.9% reporting severe or extremely severe depressive symptoms and 27.0% reporting severe anxiety symptoms, with the highest prevalence among those aged 20–24 years [24]. Furthermore, insomnia prevalence increased from the pandemic period through 2024, suggesting persistent mental health sequelae [25]. Consequently, pre-pandemic norms may no longer accurately reflect current population health.

Relying on outdated data introduces a substantial risk of bias into economic evaluations and health policy decisions. Using pre-pandemic baselines may misrepresent the cost-effectiveness of new interventions and misinform public health planning. Given the reliance on population norms for HTA and resource allocation, there is a pressing need for updated post-pandemic benchmarks.

Prior EQ-5D studies in Singapore have established important reference points. Abdin and colleagues reported a mean EQ-5D index of 0.950 in 2010 [26]. Tan and colleagues reported a mean of 0.938 using data collected in 2018, though this was based on 600 respondents aged 21 years and above who resided exclusively in public housing [20]. While many countries continue to rely on pre-pandemic norms, the combination of outdated data, limited sample coverage, and the recent availability of a Singapore-specific value set provides a compelling rationale for updated norms. Accordingly, the primary aim of this study was to generate contemporary EQ-5D-5L population norms for Singapore using data from a nationally representative household survey. This provides an updated HRQoL baseline for economic modelling, public health planning, and health equity monitoring. The secondary aim was to examine independent associations between sociodemographic and socioeconomic characteristics and HRQoL, measured using the EQ-5D-5L index and EQ VAS.

## Methods

### Study design and sampling

This study used a secondary analysis of data from a cross-sectional household survey, conducted as part of a larger project on psychological well-being [27]. The survey employed a three-stage stratified sampling design with demographic quotas for age, sex, and ethnicity to ensure population representativeness. Residential addresses were selected from a national sampling frame provided by the Singapore Department of Statistics, targeting citizens and permanent residents aged 15 to 99 years. In the first stage, residential addresses were randomly selected from the national sampling frame. In the second stage, households were contacted through multiple visit attempts. In the third stage, up to two eligible individuals per household were recruited according to demographic quotas for age, sex, and ethnicity.

Data were collected between 13 May and 30 July 2024 at participants’ homes or nearby convenient locations. Survey teams made multiple contact attempts per household, including at least one weekend visit and one weekday evening visit after 7:00 pm. When households were ineligible or unreachable, we selected replacement households from the same building or neighbourhood to maintain quota integrity.

The parent study recruited 2,006 participants from 3,173 assessed household addresses [27]. Of these, one participant was excluded owing to missing EQ-5D-5L data, yielding a final analytic sample of 2,005 respondents. All participants who completed the EQ-5D-5L in the parent survey were included; no additional exclusion criteria were applied. Although the parent study was designed to validate a psychological well-being instrument, the achieved sample of 2,005 respondents is more than adequate for generating population norms [28], exceeding the size of most published EQ-5D-5L norm studies [16–19] and providing sufficient cell sizes across major age-sex strata.

### Participants and data collection

Eligible participants were Singapore citizens or permanent residents aged 15 years or older who were able to understand and communicate in English, Chinese, Malay, or Tamil. Participants aged 65 years and above were additionally required to pass the Abbreviated Mental Test (AMT) to confirm adequate cognitive capacity. For households with two or more eligible individuals, a maximum of two were recruited, one aged below 35 years and one aged 35 years or older.

Data were collected using structured electronic questionnaires administered on digital tablets. Depending on the respondent’s preference, the survey was either self-administered or interviewer-administered in the respondent’s preferred language. Interviewers received structured training on the administration guide, informed consent procedures, and tablet-based data collection. Quality control measures included supervisory field checks, real-time electronic data validation, and regular debriefing sessions. Participants received SGD 20 in cash as compensation for their time.

### HRQoL measures

HRQoL was assessed using the EQ-5D-5L and the EQ VAS. Health utility index scores were derived by applying the most recent Singapore-specific EQ-5D-5L value set [29]. This tariff generates scores ranging from −0.851 (assigned to health state 55555, representing the worst possible health) to 1.000 (assigned to 11111, representing full health), with 0 representing a health state considered equivalent to death. Negative values indicate health states valued by the general population as worse than death, which is a well-established feature of preference-based utility measures [30]. The EQ VAS records a respondent’s overall health on the day of survey using a vertical numerical scale ranging from 0 (“the worst health you can imagine”) to 100 (“the best health you can imagine”) [31].

### Data analysis

Statistical analyses were conducted using Stata version 18.0 (StataCorp LLC, College Station, TX, USA) [32]. All statistical tests were two-tailed, with *p* < 0.05 considered statistically significant. Descriptive statistics included means and standard deviations for continuous variables and frequencies and percentages for categorical variables. These statistics summarised participant characteristics stratified by age group, gender, and ethnicity. Sampling weights were not applied. In practice, population norms are interpreted using age-by-gender-specific mean scores rather than a single overall mean. Because the normative values are stratified by demographic characteristics (Table 2 and S1), oversampling of subgroups does not bias the stratum-specific estimates. This approach is consistent with most published EQ-5D-5L population norm studies, which report unweighted descriptive statistics.

Data completeness was high. Only one respondent was excluded because of missing EQ-5D-5L data. All sociodemographic variables were fully observed. For household income, 20.1% of respondents disclosed either ‘do not know’ (11.0%) or ‘prefer not to answer’ (9.1%); these respondents were retained in all analyses with income coded as ‘Unknown’ in regression models. No imputation was performed.

Univariable analyses compared mean EQ-5D index and EQ VAS scores across sociodemographic subgroups using independent-sample t-tests for binary comparisons and one-way analysis of variance (ANOVA) for variables with more than two categories. Given the large sample size, these parametric tests are robust to violations of normality assumptions, consistent with standard practice in EQ-5D population norm studies [13, 20, 26].

To identify independent associations between sociodemographic variables and HRQoL, we specified two multivariable ordinary least squares (OLS) linear regression models, with the EQ-5D index and EQ VAS scores as dependent variables. We selected OLS regression for its interpretability and comparability with international literature. Although EQ-5D index scores are bounded and exhibit ceiling effects, OLS provides robust population-level estimates in large samples [13, 33, 34] and performs comparably to alternative models such as Tobit regression for descriptive purposes [35]. Covariates included gender, age group, ethnicity, education level, income, housing type, and marital status. Regression diagnostics were assessed, including residual plots, Q-Q plots, and variance inflation factors (VIFs) for multicollinearity. The regression analysis complements the descriptive norms by identifying independent sociodemographic predictors of HRQoL after mutual adjustment. Given the descriptive objectives of the study, we did not adjust for multiple comparisons.

### Ethics considerations

Ethical approval was obtained from the Institutional Review Board of the National University of Singapore (Reference: 2023 − 168). All participants provided written informed consent. The study was conducted in accordance with the Declaration of Helsinki.

## Results

### Sample characteristics

A total of 2,005 respondents were included in the survey. The sample was balanced by gender (51.0% female, 49.0% male) and predominantly Chinese (74.1%), with representation from Malay (13.9%), Indian (9.9%), and other ethnic groups (2.2%). Detailed sample characteristics, including educational attainment, housing type, and household income distributions, are presented in Table 1.

**Table 1:** Sociodemographic profile of study participants and representativeness compared to the 2024 Singapore population

Characteristics	Full sample		General population
	*n*	%	%
*Gender*			
Female	1023	51.0	51.3
Male	982	49.0	48.7
*Ethnicity*			
Chinese	1485	74.1	74.0
Malay	278	13.9	13.5
Indian	198	9.9	9.0
Other	44	2.2	3.4
*Education level*			
Primary or lower	209	10.4	14.5
Secondary and post-secondary	745	37.2	31.4
Tertiary	1051	52.4	54.1
*Age groups*			
15–17 years old	132	6.6	
18–24 years old	296	14.8	
25–34 years old	574	28.6	
35–44 years old	244	12.2	
45–54 years old	242	12.1	
55–64 years old	236	11.8	
65–74 years old	179	8.9	
75 years old or above	102	5.1	
*Survey Language*			
English	1753	87.4	
Chinese	239	11.9	
Malay	12	0.6	
Tamil	1	< 0.1	
*Housing type*			
HDB/JTC flat (1–2 room)	161	8.0	
HDB/JTC flat (3 room)	398	19.9	
HDB/JTC flat (4 room)	794	39.6	
HDB/JTC flat (5 room & above/Executive)	468	23.3	
Condominium/Private flat	161	8.0	
Bungalow/Semi-detached/terrace house	23	1.1	
*Household income*			
< SGD 2,000	309	15.4	
SGD 2,000–3,999	343	17.1	
SGD 4,000–5,999	318	15.9	
SGD 6,000–9,999	338	16.9	
SGD 10,000–14,999	184	9.2	
SGD 15,000 and above	109	5.4	
Do not know	221	11.0	
Prefer not to answer	183	9.1	

HDB - Housing and Development Board, a statutory board under the Ministry of National Development responsible for public housing; JTC - Jurong Town Corporation, a government agency under the Ministry of Trade and Industry responsible for industrial infrastructure

General population estimates for gender and ethnicity were obtained from the Singapore Department of Statistics *Population Trends 2024* (https://www.singstat.gov.sg/-/media/files/publications/population/population2024.pdf). Education estimates were derived from the Singapore Department of Statistics *Labour Force Survey 2024* for residents aged 25 years and over (https://tablebuilder.singstat.gov.sg/table/TS/M850701). Comparisons should be interpreted with caution because the study sample includes younger participants (15–24 years)

Population distributions for household income and housing type are not shown because national statistics use category definitions that are not directly comparable with those used in this survey

Education level was grouped into three categories: “Primary or lower” includes no formal education and completed primary school; “Secondary and post-secondary” includes lower secondary, completed secondary, ITE/ITC/NTC, and post-secondary non-tertiary (A-level/Polytechnic Foundation); “Tertiary” includes polytechnic diploma, professional qualification, university degree, and postgraduate degree. SGD 1 = USD 0.78

### EQ-5D-5L health state profiles

**Fig. 1:**
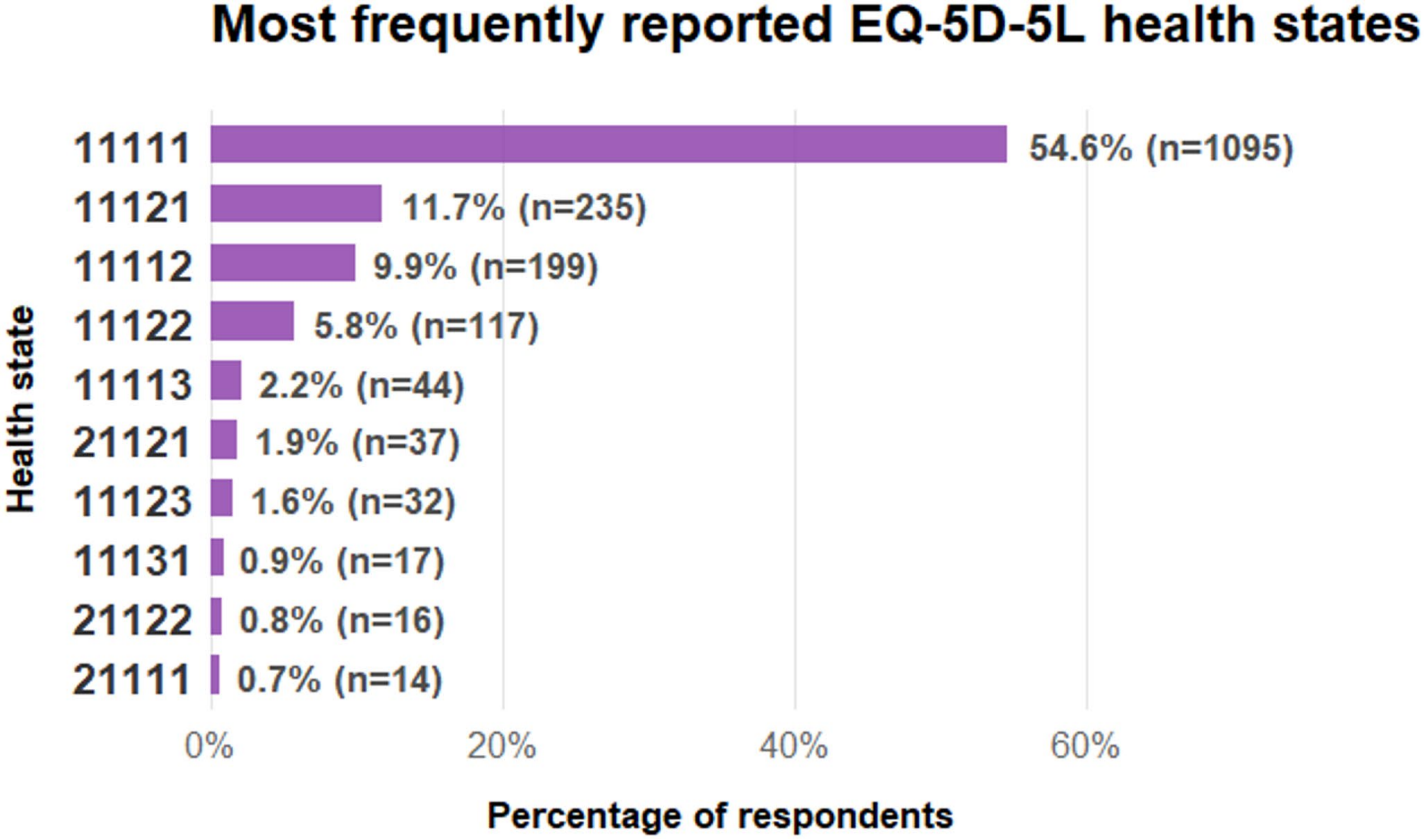
Most frequently reported EQ-5D-5L health states among respondents. This figure shows the distribution of the most common EQ-5D-5L health states reported by participants. The health state 11111, representing no problems across all five dimensions, was most frequently reported (54.6%, *n* = 1095). The remaining health states occurred much less frequently, with 11121 and 11112 accounting for 11.7% (*n* = 235) and 9.9% (*n* = 199) of responses, respectively

The health state ‘11111’, representing no problems across all five dimensions, was the most frequently reported, accounting for 54.6% of the sample (Fig. 1). Other prevalent states included ‘11121’ (slight pain/discomfort; 11.7%) and ‘11112’ (slight anxiety/depression; 9.9%). Together, the ten most common health states accounted for 90.9% of all reported profiles.

**Fig. 2:**
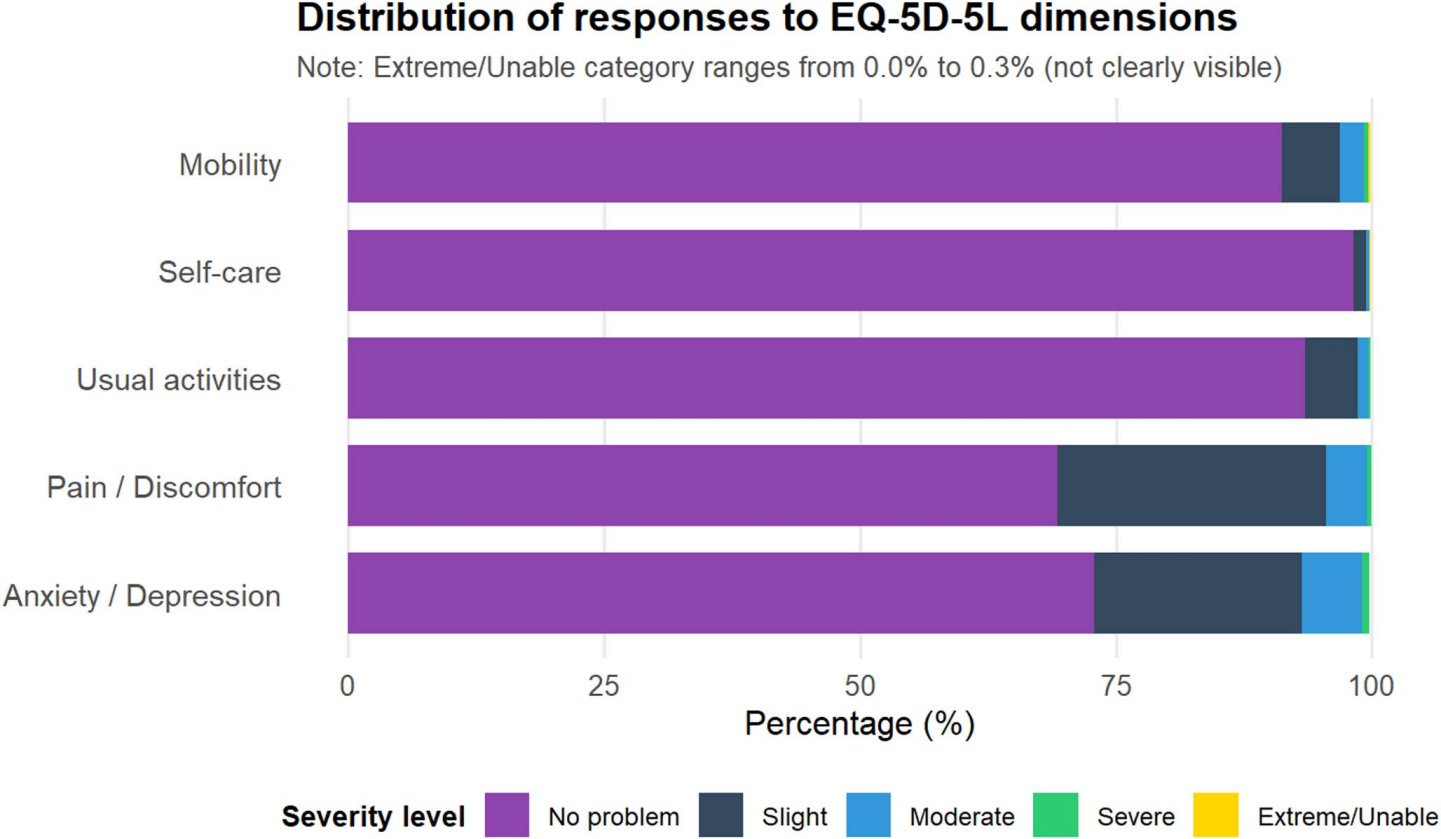
Distribution of responses across EQ-5D-5L dimensions. This figure presents the proportion of participants reporting different severity levels (no problems, slight, moderate, severe, or extreme/unable) across each EQ-5D-5L dimension: mobility, self-care, usual activities, pain/discomfort, and anxiety/depression. Most respondents reported no problems in all dimensions, with the highest proportion of issues reported in the pain/discomfort and anxiety/depression dimensions

As detailed in Fig. 2 and Table S2, functional impairments were relatively uncommon (Figures S1 and S2). Specifically, 91.2% reported no mobility limitations, 98.2% reported no self-care difficulties, and 93.5% experienced no problems with usual activities. In contrast, pain/discomfort and anxiety/depression were more prevalent. Overall, 30.7% reported any pain/discomfort (including 4.0% reporting moderate and 0.5% reporting severe pain/discomfort), and 27.1% reported any anxiety/depression (6.8% reporting moderate to extreme problems).

### Descriptive HRQoL scores

The overall sample mean was 0.933 (SD 0.122) for the EQ-5D index and 82.3 (SD 13.3) for the EQ VAS (Tables 2 and 3; Figure S3). No statistically significant gender differences were observed for either the EQ-5D index (*p* = 0.163) or EQ VAS (*p* = 0.289).

**Table 2:** Mean EQ-5D-5L index and EQ VAS scores stratified by gender, ethnicity, and age group

Variables	Measure	15–17	18–24	25–34	35–44	45–54	55–64	65–74	75+	Total
*Gender*										
Female	n	61	147	293	129	126	121	93	53	1023
	EQ-5D-5L	0.925	0.918	0.945	0.934	0.947	0.940	0.927	0.805	0.929
	EQ VAS	83.4	82.8	83.5	84.7	82.6	82.2	80.3	76.9	82.6
Male	n	71	149	281	115	116	115	86	49	982
	EQ-5D-5L	0.944	0.940	0.943	0.951	0.942	0.920	0.921	0.905	0.937
	EQ VAS	85.8	84.0	83.0	83.2	81.9	78.5	78.1	77.3	82.0
*Ethnicity*										
Chinese	n	86	206	421	177	181	178	148	88	1485
	EQ-5D-5L	0.930	0.935	0.950	0.952	0.951	0.940	0.924	0.867	0.938
	EQ VAS	84.4	82.4	83.0	83.3	81.8	80.1	78.0	76.7	81.7
Malay	n	21	51	92	31	25	32	19	7	278
	EQ-5D-5L	0.924	0.889	0.936	0.889	0.892	0.921	0.936	0.779	0.912
	EQ VAS	83.3	82.9	83.5	83.9	81.0	85.0	86.9	79.0	83.5
Indian	n	23	34	51	26	28	20	10	6	198
	EQ-5D-5L	0.980	0.948	0.909	0.935	0.941	0.853	0.893	0.717	0.920
	EQ VAS	88.8	89.0	83.3	86.2	85.2	74.2	80.7	82.8	84.5
Other	n	2	5	10	10	8	6	2	1	44
	EQ-5D-5L	0.772	0.983	0.961	0.954	0.989	0.948	0.978	1.000	0.958
	EQ VAS	65.0	88.6	88.1	90.8	87.2	86.8	90.0	65.0	87.0
Total	n	132	296	574	244	242	236	179	102	2005
	EQ-5D-5L	0.935	0.929	0.944	0.942	0.945	0.930	0.924	0.853	0.933
	EQ VAS	84.7	83.4	83.2	84.0	82.3	80.4	79.2	77.1	82.3

EQ-5D-5L index values were calculated using the Singapore EQ-5D-5L value set. EQ VAS scores range from 0 (worst imaginable health) to 100 (best imaginable health). Results for the “Others” ethnic group should be interpreted with caution due to small sample sizes across age groups (*n* = 1–10)

**Table 3:** Bivariate analysis of EQ-5D-5L index and EQ VAS scores by sociodemographic characteristics

Variables	*n*	EQ-5D-5L index			EQ VAS		
		Mean	SD	*p*-value	Mean	SD	*p*-value
Full sample	2005	0.933	0.122		82.3	13.3	
*Gender*							
Female	1023	0.929	0.124	0.163	82.6	13.5	0.289
Male	982	0.937	0.119		82.0	13.2	
*Age group*							
15–17	132	0.935	0.124	< 0.001***	84.7	15.5	< 0.001***
18–24	296	0.929	0.112		83.4	14.0	
25–34	574	0.944	0.091		83.2	13.0	
35–44	244	0.942	0.124		84.0	12.1	
45–54	242	0.945	0.109		82.3	11.9	
55–64	236	0.930	0.132		80.4	14.0	
65–74	179	0.924	0.128		79.2	12.6	
≥ 75	102	0.853	0.218		77.1	13.7	
*Ethnicity*							
Chinese	1485	0.938	0.107	0.001**	81.7	13.0	0.001**
Malay	278	0.912	0.165		83.5	14.5	
Indian	198	0.920	0.149		84.5	13.7	
Other	44	0.958	0.089		87.0	12.2	
*Survey language*							
English	1753	0.933	0.120	0.126	82.7	13.2	< 0.001***
Chinese	239	0.932	0.131		79.3	14.3	
Malay	12	0.958	0.073		87.5	7.4	
Tamil	1	0.656	—		51.0	—	
*Education level*							
Primary or lower	209	0.905	0.176	< 0.001***	80.5	14.9	0.056
Secondary and post-secondary	745	0.923	0.140		82.1	14.3	
Tertiary	1051	0.946	0.089		82.8	12.2	
*Household income*							
< SGD 2,000	309	0.890	0.189	< 0.001***	80.2	14.7	< 0.001***
SGD 2,000–3,999	343	0.940	0.100		82.8	13.4	
SGD 4,000–5,999	318	0.941	0.097		82.6	13.7	
SGD 6,000–9,999	338	0.949	0.086		83.6	12.1	
SGD 10,000–14,999	184	0.954	0.080		85.0	10.3	
SGD 15,000+	109	0.953	0.066		83.4	9.2	
Do not know	221	0.926	0.131		81.3	14.6	
Prefer not to answer	183	0.927	0.139		80.2	14.7	
*Housing type*							
HDB/JTC flat (1–2 room)	161	0.872	0.197	< 0.001***	81.1	15.5	0.883
HDB/JTC flat (3 room)	398	0.937	0.104		82.6	13.5	
HDB/JTC flat (4 room)	794	0.939	0.112		82.3	13.3	
HDB/JTC flat (5 room & above/Executive)	468	0.936	0.120		82.4	12.9	
Condominium/Private flat	161	0.945	0.101		82.8	12.0	
Bungalow/Semi-detached/terrace house	23	0.954	0.073		83.0	11.3	

EQ-5D-5L index values were calculated using the Singapore EQ-5D-5L value set. EQ VAS scores range from 0 (worst imaginable health) to 100 (best imaginable health). P-values were derived from independent samples t-tests (gender) or one-way ANOVA (all other variables). SD could not be computed for Tamil (*n* = 1). SGD 1 = USD 0.78. *** *p* < 0.001, ** *p* < 0.01, * *p* < 0.05

Both EQ-5D index and EQ VAS scores differed significantly across age categories (*p* < 0.001). Index scores were highest among respondents aged 25–54 years (mean range: 0.942–0.945), while respondents aged ≥ 75 years reported the lowest scores (mean: 0.853). A similar pattern was observed for EQ VAS scores, which ranged from 84.7 among respondents aged 15–17 years to 77.1 among the oldest age group.

Significant differences across ethnic groups were observed for both measures (*p* = 0.001; Figure S4). Malay respondents reported the lowest EQ-5D index scores (0.912), while respondents classified as ‘Other’ reported the highest scores (0.958). For the EQ VAS, Indian respondents reported the highest mean scores (84.5), whereas Chinese respondents reported the lowest (81.7).

EQ-5D index scores differed significantly across education categories (*p* < 0.001), with higher scores observed among respondents with tertiary education. This association was marginal for EQ VAS scores (*p* = 0.056). Both outcomes differed significantly across income categories (*p* < 0.001). Housing type was significantly associated with EQ-5D index scores (*p* < 0.001) but not with EQ VAS scores (*p* = 0.883).

### Factors associated with HRQoL Scores

Multivariable regression results are presented in Table 4. For the EQ-5D index, compared with the reference group (aged 15–17 years), older age was significantly associated with lower scores. The largest negative association was observed among respondents aged ≥ 75 years (β = −0.083, 95% CI −0.120 to −0.046). Relative to reference categories (income < SGD 2,000; 1–2 room HDB flat; no formal education), higher income levels, larger housing types, and higher educational attainment were independently associated with higher EQ-5D index scores.

**Table 4:** Multivariable linear regression of sociodemographic factors associated with EQ-5D-5L index and EQ VAS scores

Variables	EQ-5D-5L index			EQ VAS		
	Adjusted β	95% CI	*p*-value	Adjusted β	95% CI	*p*-value
(Intercept)	0.870	0.833, 0.906	< 0.001***	82.3	78.2, 86.4	< 0.001***
*Gender (Ref: Male)*						
Female	−0.006	−0.017, 0.004	0.243	0.7	−0.5, 1.8	0.258
*Age group (Ref: 15–17)*						
18–24	−0.007	−0.033, 0.019	0.577	−1.5	−4.4, 1.4	0.317
25–34	−0.006	−0.033, 0.021	0.678	−2.5	−5.5, 0.5	0.107
35–44	−0.018	−0.049, 0.013	0.248	−2.7	−6.2, 0.7	0.120
45–54	−0.012	−0.043, 0.019	0.451	−4.4	−7.8, − 0.9	0.013*
55–64	−0.019	−0.049, 0.011	0.211	−6.0	−9.3, − 2.6	< 0.001***
65–74	−0.016	−0.047, 0.016	0.335	−6.3	−9.8, − 2.8	< 0.001***
≥ 75	−0.083	−0.120, − 0.046	< 0.001***	−8.0	−12.1, − 3.9	< 0.001***
*Ethnicity (Ref: Chinese)*						
Malay	−0.017	−0.034, − 0.001	0.040*	1.8	−0.0, 3.6	0.055
Indian	−0.016	−0.034, 0.002	0.086	2.5	0.5, 4.5	0.014*
Others	0.019	−0.016, 0.055	0.291	4.7	0.7, 8.6	0.021*
*Education level (Ref: Primary or lower)*						
Secondary and post-secondary	0.004	−0.015, 0.023	0.691	−0.2	−2.3, 2.0	0.889
Tertiary	0.006	−0.016, 0.028	0.599	−0.8	−3.2, 1.7	0.545
*Household income (Ref: < SGD 2*,*000)*						
SGD 2,000–3,999	0.032	0.013, 0.051	0.001**	1.3	−0.9, 3.4	0.243
SGD 4,000–5,999	0.025	0.004, 0.045	0.020*	0.8	−1.5, 3.1	0.504
SGD 6,000–9,999	0.028	0.007, 0.049	0.008**	2.0	−0.3, 4.4	0.090
SGD 10,000–14,999	0.031	0.006, 0.055	0.015*	3.1	0.3, 5.8	0.027*
SGD 15,000+	0.030	0.001, 0.058	0.043*	1.7	−1.5, 4.9	0.303
Unknown	0.020	0.001, 0.038	0.043*	−0.9	−3.0, 1.2	0.411
*Marital status (Ref: Cohabiting with partner)*						
Never married, no partner	0.010	−0.007, 0.028	0.236	−0.0	−1.9, 1.9	0.991
Married	0.032	0.013, 0.051	0.001**	1.4	−0.7, 3.5	0.194
Separated	−0.004	−0.087, 0.080	0.934	−10.3	−19.6, − 1.0	0.030*
Widowed	−0.010	−0.045, 0.026	0.598	0.5	−3.5, 4.5	0.815
Divorced	−0.016	−0.047, 0.015	0.311	2.4	−1.1, 5.8	0.184
*Housing type (Ref: HDB/JTC flat 1–2 room)*						
HDB/JTC flat (3 room)	0.049	0.026, 0.072	< 0.001***	1.6	−0.9, 4.2	0.206
HDB/JTC flat (4 room)	0.043	0.021, 0.065	< 0.001***	1.2	−1.3, 3.6	0.345
HDB/JTC flat (5 room & above/Executive)	0.038	0.015, 0.062	0.001**	1.4	−1.2, 4.1	0.286
Condominium/Private flat	0.043	0.015, 0.072	0.003**	1.9	−1.3, 5.1	0.247
Bungalow/Semi-detached/terrace house	0.064	0.011, 0.117	0.019*	3.4	−2.6, 9.3	0.266

“Unknown” income combines respondents who answered “Do not know” or “Prefer not to answer”. CI — confidence interval. SGD 1 = USD 0.78. *** *p* < 0.001, ** *p* < 0.01, * *p* < 0.05

For the EQ VAS, compared with the youngest age group (15–17 years), significantly lower scores were observed from age 45–54 years onwards. Indian (β = 2.5, 95% CI 0.5 to 4.5) and ‘Other’ (β = 4.7, 95% CI 0.7 to 8.6) respondents reported significantly higher EQ VAS scores than Chinese respondents (reference category). Monthly household income of SGD 10,000–14,999 was positively associated with EQ VAS scores (β = 3.1, 95% CI 0.3 to 5.8). Respondents who were separated reported significantly lower EQ VAS scores (β = −10.3, 95% CI −19.6 to −1.0), although this subgroup was small.

Regression diagnostics confirmed approximate linearity and acceptable homoscedasticity. All VIF values were below the conventional threshold of 10 (mean VIF = 2.56; highest VIF = 5.62), indicating acceptable levels of multicollinearity (Table S4).

## Discussion

### Main findings

This study provides the first nationally representative, post-pandemic EQ-5D-5L population norms for Singapore. The general population reported high HRQoL, with a mean EQ-5D index of 0.933 and a mean EQ VAS score of 82.3. The health state distribution was heavily skewed towards minimal impairment, with over half of respondents (54.6%) reporting perfect health (‘11111’). This proportion was highest among respondents aged 15–17 years (60.6%) and declined with advancing age. The overall mean EQ-5D index was 0.950 in 2010 (Abdin et al. [26], EQ-5D-3L), lower at 0.938 in 2018 (Tan et al. [20], EQ-5D-5L with crosswalk) and 0.933 in the present study (EQ-5D-5L with Singapore value set). Direct numerical comparisons should be interpreted with caution owing to differences in instruments, value sets, sample age ranges, and coverage. A detailed, structured comparison of pre-pandemic and post-pandemic population health in Singapore, including subgroup-level analyses, is presented in a companion paper [36].

Despite high overall health status, significant domain-specific burdens emerged. The most prevalent problems were pain/discomfort (30.7%) and anxiety/depression (27.1%), while functional limitations remained rare. This pattern of high functional health coexisting with substantial psychosocial and pain-related morbidity suggests that the post-pandemic health landscape may be characterised not by functional disability, but by less visible burdens of mental distress and chronic pain. This finding is consistent with international evidence documenting elevated anxiety and depression following COVID-19 [37–39].

As anticipated, respondents aged 15–24 years reported the highest prevalence of anxiety/depression (35.7%), compared with 24.0% among those aged 25 years and above. This suggests that inclusion of younger respondents partly accounts for the higher overall prevalence relative to earlier Singapore studies [20, 26]. However, this pattern may also reflect generational differences in willingness to report psychological symptoms [40]. Younger cohorts may be more open to acknowledging mental distress due to reduced stigma and greater mental health literacy [41, 42], rather than experiencing higher prevalence alone. If respondents systematically underreport anxiety or depression, the population norms presented here may overestimate true HRQoL. Practitioners using these norms as reference values should therefore interpret small observed decrements with appropriate caution, as they may represent larger true differences [40]. These findings align with Singapore’s recognition of youth mental health as a national priority [43], supported by evidence showing that 25.3% of individuals aged 18–29 years experienced poor mental health in 2022 (up from 21.5% in 2020), and that suicide remained the leading cause of death among those aged 10–29 years [44, 45].

Pain/discomfort showed distinct age-related patterns. Prevalence was lowest among younger respondents (15–24 years: 21.0%), intermediate among working-age adults (25–54 years: 26.9%), and highest among older adults (≥ 65 years: 53.7%). While the high prevalence among older adults is consistent with age-related musculoskeletal conditions and chronic disease burden, the finding that more than one-quarter of working-age adults reported pain may reflect pandemic-era lifestyle changes, including changes in physical activity patterns [46, 47]. These results support age-targeted interventions, including mental health support for youth, workplace health promotion for working adults, and pain management strategies for older populations.

Regression analyses demonstrated marked socioeconomic gradients in HRQoL. Higher household income, higher educational attainment, and residence in larger housing were independently associated with higher EQ-5D index scores, consistent with international and local evidence [48–51]. These gradients persist even in a setting with universal health coverage and likely operate through multiple pathways, including material resources (such as nutrition, housing quality, and access to health-promoting activities) [52], psychosocial advantages (including lower chronic stress, greater perceived control, and stronger social capital) [53], and differences in health literacy [54]. Population health strategies should therefore address upstream social and economic determinants alongside biomedical interventions.

Age-related associations with HRQoL revealed an important distinction [49, 51, 55, 56]. Compared to the youngest reference group (15–17 years), EQ VAS scores were significantly lower starting from age 45–54, while the EQ-5D index showed its sharpest decline only among those aged 75 years and above. This divergence reflects the different constructs captured by these measures. The EQ-5D index represents preference-weighted functional health derived from general population value sets, while the EQ VAS captures respondents’ own global assessment of their health status. As a result, perceived health decline may emerge earlier than severe functional impairment, likely driven by accumulating chronic but non-disabling conditions [57]. Furthermore, the EQ VAS may capture health dimensions beyond those included in the EQ-5D-5L descriptive system, such as fatigue, vitality, and sleep quality, which may contribute to perceived health decline in middle age [58]. In addition, ceiling effects may contribute to this pattern. In our sample, 54.6% reported the ceiling state ‘11111’, constraining the EQ-5D index at 1.0 and limiting its ability to discriminate among individuals in perfect functional health, whereas the EQ VAS continues to capture variation in perceived health. Moreover, the EQ-5D index may not operate as a fully interval scale, with changes at lower levels of health representing larger perceived differences than equivalent changes near the ceiling [59]. Together, these findings support a life-course approach to health service planning, with preventive and well-being initiatives targeting middle-aged populations before functional decline becomes evident.

Ethnic patterns revealed nuanced findings. Malay respondents reported the lowest EQ-5D index scores, while Indian respondents reported the highest EQ VAS scores. This pattern is consistent with prior evidence that health outcomes and health perceptions vary across ethnic groups [60–62]. The divergence between index and VAS scores across ethnic groups may reflect unmeasured structural factors, cultural differences in conceptualising health [63–65], or measurement artefacts. Notably, formal measurement invariance testing of the EQ-5D across ethnic groups in Singapore has not been conducted. Future research should examine whether these differences reflect true health disparities or systematic differences in response styles.

The absence of statistically significant gender differences contrasts with international literature, in which women typically report lower HRQoL [7, 49, 51]. However, prior EQ-5D studies in Singapore have reported inconsistent gender patterns [20, 26], suggesting that this finding may not be specific to the post-pandemic period. Further research is needed to understand the social, cultural, and contextual factors that may contribute to apparent gender parity in HRQoL in Singapore.

### Strengths and limitations

This study’s large, nationally representative sample provides robust post-pandemic population norms. However, several limitations should be acknowledged. First, the cross-sectional design permits only associational inference. Second, the use of demographic quotas introduces a non-probability element into the sampling design, which may limit strict inferential generalisability. Nevertheless, the random selection of addresses from the national sampling frame and the close concordance between the sample and census distributions (Table 1) suggest that the achieved sample reasonably approximates the Singapore resident population. Third, sampling weights were not applied. However, because population norms are derived from subgroup-specific values rather than a single overall mean, differential sampling fractions across strata are unlikely to bias the normative estimates. Fourth, while most participants completed the questionnaire themselves (82.2%), a smaller proportion were interviewer-administered (11.2%) or used a mixed approach (6.6%). All interviewers followed a standardised administration protocol, and the questionnaire was presented on a consistent digital tablet format across both modes, minimising potential mode effects. As interviewer presence may nonetheless influence responses to sensitive questions, this has been noted as a limitation. Fifth, cognitive screening using the AMT was applied only to respondents aged 65 years and above. This approach may have excluded cognitively impaired older adults and may therefore have led to a modest overestimation of HRQoL in this age group. Sixth, HRQoL measures were self-reported and may be subject to social desirability and recall bias. Seventh, some subgroups were too small for meaningful analysis (for example, Tamil-speaking respondents, *n* = 1). Eighth, 9.1% of respondents declined to report income. This non-random missingness may lead to underestimation of socioeconomic gradients. However, housing type, which was significantly associated with EQ-5D index scores, may partially capture socioeconomic position for these individuals. Finally, institutionalised populations were excluded; therefore, the findings reflect community-dwelling residents only.

### Implications for policy and practice

These post-pandemic population norms provide an essential and updated reference for cost-utility analyses and health technology assessments in Singapore. Using contemporary values ensures that economic evaluations more accurately reflect current population health and potential utility gains. From a public health perspective, the coexistence of high functional health with a substantial burden of mental health problems and pain highlights the need to extend interventions beyond clinical settings. Addressing social determinants through community-based mental health services, workplace health promotion, and strategies to reduce socioeconomic inequality will be critical.

### Future research

Three priorities for future research emerge. First, longitudinal studies are needed to determine whether elevated anxiety and pain prevalence represents a persistent shift or a transient post-pandemic effect, and to clarify causal pathways linking socioeconomic status and health trajectories. Second, qualitative and mixed-methods research should explore ethnic and gender differences in HRQoL to inform culturally appropriate interventions; this should include formal measurement invariance testing of the EQ-5D across ethnic groups in Singapore. Third, linking HRQoL data with clinical records would enable assessment of concordance between self-reported health and objective morbidity, informing intervention design for pain/discomfort and anxiety/depression.

## Conclusion

This study establishes updated, post-pandemic EQ-5D-5L population norms for Singapore. These norms provide an essential benchmark for interpreting EQ-5D-5L scores and quantifying health burden in cost-utility analyses, while identifying key priorities for public health action and for addressing socioeconomic health inequalities.

## Supplementary Information

Below is the link to the electronic supplementary material.

Supplementary Material 1

## Data Availability

The data generated and analysed during the current study are not publicly available due to ethical and privacy restrictions but are available from the corresponding author upon reasonable request and with approval from the Institutional Review Board of the National University of Singapore.
