# Supplementary material for: EQ-5D-5L population norms for Singapore: a household survey-based analysis
Source: Qual Life Res. 2026 Jun 6;35(7):181. doi: 10.1007/s11136-026-04272-2 (PMC13242447; doi:10.1007/s11136-026-04272-2)
Supplement: Supplementary file 1 — Supplementary Material 1 [file 11136_2026_4272_MOESM1_ESM.docx]

**Table S1. EQ-5D-5L index, EQ VAS scores, and percentage reporting any problems by demographic characteristics, with 95% confidence intervals**

| **Characteristic** | **Category** | **n** | **EQ-5D-5L index**  **(95% CI)** | **EQ VAS (95% CI)** | **Any problems % (95% CI)** |
| --- | --- | --- | --- | --- | --- |
| **Age group** | 15-17 | 132 | 0.935 (0.914, 0.956) | 84.7 (82.1, 87.4) | 39.4 (31.0, 48.3) |
|  | 18-24 | 296 | 0.929 (0.916, 0.942) | 83.4 (81.8, 85.0) | 47.0 (41.2, 52.8) |
|  | 25-34 | 574 | 0.944 (0.937, 0.951) | 83.2 (82.1, 84.3) | 43.2 (39.1, 47.4) |
|  | 35-44 | 244 | 0.942 (0.926, 0.958) | 84.0 (82.5, 85.5) | 40.2 (34.0, 46.6) |
|  | 45-54 | 242 | 0.945 (0.931, 0.958) | 82.3 (80.8, 83.8) | 42.1 (35.9, 48.6) |
|  | 55-64 | 236 | 0.930 (0.913, 0.947) | 80.4 (78.6, 82.2) | 45.3 (38.9, 51.9) |
|  | 65-74 | 179 | 0.924 (0.906, 0.943) | 79.2 (77.4, 81.1) | 57.0 (49.4, 64.3) |
|  | 75+ | 102 | 0.853 (0.810, 0.896) | 77.1 (74.4, 79.8) | 60.8 (50.6, 70.3) |
| **Gender** | Female | 1023 | 0.929 (0.922, 0.937) | 82.6 (81.8, 83.5) | 47.3 (44.2, 50.4) |
|  | Male | 982 | 0.937 (0.930, 0.944) | 82.0 (81.2, 82.8) | 43.4 (40.3, 46.5) |
| **Ethnicity** | Chinese | 1485 | 0.938 (0.933, 0.944) | 81.7 (81.0, 82.3) | 45.1 (42.6, 47.7) |
|  | Malay | 278 | 0.912 (0.892, 0.931) | 83.5 (81.8, 85.2) | 52.2 (46.1, 58.2) |
|  | Indian | 198 | 0.920 (0.899, 0.941) | 84.5 (82.6, 86.4) | 41.9 (35.0, 49.1) |
|  | Others | 44 | 0.958 (0.931, 0.986) | 87.0 (83.3, 90.7) | 27.3 (15.0, 42.8) |
| **Ethnicity – Gender** | Chinese – Female | 763 | 0.935 (0.927, 0.942) | 82.3 (81.3, 83.2) | – |
|  | Chinese – Male | 722 | 0.942 (0.934, 0.950) | 81.1 (80.1, 82.0) | – |
|  | Malay – Female | 138 | 0.914 (0.885, 0.943) | 83.0 (80.4, 85.5) | – |
|  | Malay – Male | 140 | 0.909 (0.883, 0.936) | 84.0 (81.7, 86.3) | – |
|  | Indian – Female | 99 | 0.903 (0.872, 0.935) | 83.8 (80.8, 86.9) | – |
|  | Indian – Male | 99 | 0.936 (0.908, 0.964) | 85.2 (82.8, 87.6) | – |
|  | Others – Female | 23 | 0.957 (0.914, 1.001) | 88.0 (82.3, 93.8) | – |
|  | Others – Male | 21 | 0.960 (0.924, 0.995) | 85.8 (80.8, 90.7) | – |
| **Total** |  | 2005 | 0.933 (0.928, 0.938) | 82.3 (81.7, 82.9) | 45.4 (43.2, 47.6) |
| 95% CIs for EQ-5D-5L index and EQ VAS were computed using normal-approximation confidence intervals; 95% CIs for percentage with any problems were computed using exact binomial (Clopper–Pearson) confidence intervals. For the Others–Female subgroup (n = 23), the upper bound of the EQ-5D-5L index CI slightly exceeds 1.0 due to the small sample size. Any problems % was not computed for ethnicity–gender subgroups as it was not reported in the original table. – = not applicable. | | | | | |
|  |  |  |  |  |  |

**Table S2. Distribution of responses to EQ-5D-5L dimensions with standard errors.**

| **Dimension** | **Level** | **n** | **%** | **SE** |
| --- | --- | --- | --- | --- |
| **Mobility** | No problem | 1829 | 91.2 | 0.63 |
|  | Slight problem | 115 | 5.7 | 0.52 |
|  | Moderate problem | 46 | 2.3 | 0.33 |
|  | Severe problem | 11 | 0.5 | 0.16 |
|  | Unable | 4 | 0.2 | 0.10 |
| **Self-care** | No problem | 1969 | 98.2 | 0.30 |
|  | Slight problem | 24 | 1.2 | 0.24 |
|  | Moderate problem | 8 | 0.4 | 0.14 |
|  | Severe problem | 1 | 0.0 | 0.05 |
|  | Unable | 3 | 0.1 | 0.09 |
| **Usual activities** | No problem | 1874 | 93.5 | 0.55 |
|  | Slight problem | 103 | 5.1 | 0.49 |
|  | Moderate problem | 22 | 1.1 | 0.23 |
|  | Severe problem | 3 | 0.1 | 0.09 |
|  | Unable | 3 | 0.1 | 0.09 |
| **Pain / Discomfort** | No pain/discomfort | 1389 | 69.3 | 1.03 |
|  | Slight | 525 | 26.2 | 0.98 |
|  | Moderate | 81 | 4.0 | 0.44 |
|  | Severe | 10 | 0.5 | 0.16 |
|  | Extreme | 0 | 0.0 | 0.00 |
| **Anxiety / Depression** | Not anxious/depressed | 1461 | 72.9 | 0.99 |
|  | Slightly | 407 | 20.3 | 0.90 |
|  | Moderately | 117 | 5.8 | 0.52 |
|  | Severely | 15 | 0.7 | 0.19 |
|  | Extremely | 5 | 0.2 | 0.11 |
| *N = 2,005. Standard errors were calculated as SE = √[p(1 – p) / N], where p is the observed proportion. Percentages may not sum to 100.0% due to rounding.* | | | | |

**Table S3. Percentage reporting any problems (levels 2-5) by EQ-5D-5L dimension, age group, and gender**

| **Age group** | **Gender** | **n** | **Mo (%)** | **SC (%)** | **UA (%)** | **PD (%)** | **AD (%)** |
| --- | --- | --- | --- | --- | --- | --- | --- |
| 15-17 | Female | 61 | 4.9 | 3.3 | 8.2 | 27.9 | 36.1 |
| 15-17 | Male | 71 | 1.4 | 1.4 | 5.6 | 15.5 | 22.5 |
| 15-17 | Total | 132 | 3.0 | 2.3 | 6.8 | 21.2 | 28.8 |
| 18-24 | Female | 147 | 6.1 | 0.0 | 8.8 | 22.4 | 48.3 |
| 18-24 | Male | 149 | 4.7 | 0.7 | 5.4 | 19.5 | 29.5 |
| 18-24 | Total | 296 | 5.4 | 0.3 | 7.1 | 20.9 | 38.9 |
| 25-34 | Female | 293 | 3.8 | 1.0 | 4.8 | 24.2 | 31.7 |
| 25-34 | Male | 281 | 5.7 | 0.7 | 4.6 | 24.6 | 31.7 |
| 25-34 | Total | 574 | 4.7 | 0.9 | 4.7 | 24.4 | 31.7 |
| 35-44 | Female | 129 | 7.0 | 3.9 | 6.2 | 28.7 | 24.0 |
| 35-44 | Male | 115 | 6.1 | 1.7 | 5.2 | 27.0 | 21.7 |
| 35-44 | Total | 244 | 6.6 | 2.9 | 5.7 | 27.9 | 23.0 |
| 45-54 | Female | 126 | 6.3 | 0.8 | 2.4 | 34.9 | 22.2 |
| 45-54 | Male | 116 | 8.6 | 0.0 | 1.7 | 28.4 | 25.9 |
| 45-54 | Total | 242 | 7.4 | 0.4 | 2.1 | 31.8 | 24.0 |
| 55-64 | Female | 121 | 11.6 | 2.5 | 6.6 | 38.8 | 12.4 |
| 55-64 | Male | 115 | 12.2 | 2.6 | 10.4 | 37.4 | 26.1 |
| 55-64 | Total | 236 | 11.9 | 2.5 | 8.5 | 38.1 | 19.1 |
| 65-74 | Female | 93 | 20.4 | 1.1 | 5.4 | 57.0 | 15.1 |
| 65-74 | Male | 86 | 14.0 | 3.5 | 5.8 | 45.3 | 17.4 |
| 65-74 | Total | 179 | 17.3 | 2.2 | 5.6 | 51.4 | 16.2 |
| 75+ | Female | 53 | 47.2 | 13.2 | 34.0 | 66.0 | 32.1 |
| 75+ | Male | 49 | 22.4 | 4.1 | 14.3 | 49.0 | 8.2 |
| 75+ | Total | 102 | 35.3 | 8.8 | 24.5 | 57.8 | 20.6 |
| All ages | Female | 1023 | 9.6 | 2.2 | 7.2 | 32.9 | 28.4 |
| All ages | Male | 982 | 7.9 | 1.4 | 5.8 | 28.4 | 25.8 |
| All ages | Total | 2005 | 8.8 | 1.8 | 6.5 | 30.7 | 27.1 |
| Any problems were defined as reporting level 2 (slight problems) or above on each EQ-5D-5L dimension. Percentages are based on the unweighted sample within each age-gender stratum. Mo - Mobility; SC - Self-care; UA - Usual activities; PD - Pain or discomfort; AD - Anxiety or depression. | | | | | | | |

**Table S4. Variance Inflation Factors for EQ-5D-5L Index and EQ VAS Regression Models**

| **Variable** | **EQ-5D-5L Index VIF** | **EQ-5D-5L Index Tolerance** | **EQ VAS VIF** | **EQ VAS Tolerance** |
| --- | --- | --- | --- | --- |
| Female | 1.03 | 0.968 | 1.03 | 0.968 |
| **Age group** |  |  |  |  |
| 18-24 years | 3.17 | 0.315 | 3.17 | 0.315 |
| 25-34 years | 5.62 | 0.178 | 5.62 | 0.178 |
| 35-44 years | 3.84 | 0.261 | 3.84 | 0.261 |
| 45-54 years | 3.78 | 0.264 | 3.78 | 0.264 |
| 55-64 years | 3.50 | 0.286 | 3.50 | 0.286 |
| 65-74 years | 3.05 | 0.328 | 3.05 | 0.328 |
| 75+ years | 2.44 | 0.410 | 2.44 | 0.410 |
| **Ethnicity** |  |  |  |  |
| Malay | 1.22 | 0.823 | 1.22 | 0.823 |
| Indian | 1.07 | 0.933 | 1.07 | 0.933 |
| Others | 1.02 | 0.982 | 1.02 | 0.982 |
| **Education level** |  |  |  |  |
| Secondary and post-secondary | 3.27 | 0.306 | 3.27 | 0.306 |
| Tertiary | 4.55 | 0.220 | 4.55 | 0.220 |
| **Monthly household income** |  |  |  |  |
| SGD 2,000-3,999 | 1.96 | 0.510 | 1.96 | 0.510 |
| SGD 4,000-5,999 | 2.15 | 0.464 | 2.15 | 0.464 |
| SGD 6,000-9,999 | 2.33 | 0.429 | 2.33 | 0.429 |
| SGD 10,000-14,999 | 1.89 | 0.529 | 1.89 | 0.529 |
| SGD 15,000+ | 1.60 | 0.626 | 1.60 | 0.626 |
| Unknown | 2.16 | 0.464 | 2.16 | 0.464 |
| **Marital status** |  |  |  |  |
| Never married, no partner | 2.26 | 0.442 | 2.26 | 0.442 |
| Married | 3.30 | 0.303 | 3.30 | 0.303 |
| Separated | 1.04 | 0.962 | 1.04 | 0.962 |
| Widowed | 1.74 | 0.576 | 1.74 | 0.576 |
| Divorced | 1.56 | 0.641 | 1.56 | 0.641 |
| **Housing type** |  |  |  |  |
| HDB/JTC flat (3 room) | 3.12 | 0.320 | 3.12 | 0.320 |
| HDB/JTC flat (4 room) | 4.35 | 0.230 | 4.35 | 0.230 |
| HDB/JTC flat (5 room & above/Executive) | 3.75 | 0.267 | 3.75 | 0.267 |
| Condominium/Private flat | 2.25 | 0.444 | 2.25 | 0.444 |
| Bungalow/Semi-detached/Terrace house | 1.20 | 0.831 | 1.20 | 0.831 |
| **Mean VIF** | **2.56** |  | **2.56** |  |
| VIF = variance inflation factor. VIF values assess multicollinearity among independent variables. VIF values are identical for both models as they include the same predictor variables (n=2,004). Reference categories: Male, 15-17 years, Chinese, Primary/Below/No formal education, Income <SGD 2,000, Never married with partner, HDB/JTC flat (1-2 room). All VIF values are below the conventional threshold of 10, indicating acceptable levels of multicollinearity. The highest VIF of 5.62 was observed for the 25-34 age group. | | | | |

**Figure S1. Proportion reporting full health (EQ-5D-5L health state 11111) by age group and gender**

*The proportion of respondents reporting full health, defined as an EQ-5D-5L health state of 11111, is shown by age group and gender. Points represent observed proportions within each age group. Blue solid lines with circles denote males, and red dashed lines with triangles denote females.*

**Figure S2. Percentage reporting any problems by EQ-5D-5L dimension and ethnicity**

*The percentage of respondents reporting any problems (levels 2–5) across the five EQ-5D-5L dimensions is shown by ethnicity. Bars represent the proportion reporting at least one problem in each dimension.*

**Figure S3. Mean EQ-5D-5L index and EQ VAS scores by age group and gender**

*Mean EQ-5D-5L index scores (top panel) and EQ visual analogue scale (EQ VAS) scores (bottom panel) are shown by age group and gender. Points represent mean values, and vertical error bars indicate 95% confidence intervals. Blue solid lines with circles denote males, and red dashed lines with triangles denote females.*

**Figure S4. Mean EQ-5D-5L index and EQ VAS scores by age group and ethnicity**

*Mean EQ-5D-5L index scores (top panel) and EQ visual analogue scale (EQ VAS) scores (bottom panel) are shown by age group and ethnicity. Points represent mean values for each age–ethnicity group.*
